# Obesity Parameters as Predictor of Poor Outcomes in Hospitalized Patients with Confirmed Mild-to-Moderate COVID-19

**DOI:** 10.3390/idr16050071

**Published:** 2024-09-12

**Authors:** Nadya R. V. Barus, Dicky Levenus Tahapary, Farid Kurniawan, Robert Sinto, Syahidatul Wafa, Wismandari Wisnu, Arif Mansjoer, Calysta Nadya Wijaya, Immanuel Felix, Tri Juli Edi Tarigan, Dante Saksono Harbuwono, Pradana Soewondo

**Affiliations:** 1 Department of Internal Medicine, Faculty of Medicine Universitas Indonesia, Jakarta 10430, Indonesia; nadyabarus@gmail.com; 2 Division of Endocrinology, Metabolism, and Diabetes, Department of Internal Medicine, Faculty of Medicine Universitas Indonesia, Dr. Cipto Mangunkusumo National Referral Hospital, Jakarta 10430, Indonesia; farid.kurniawan01@ui.ac.id (F.K.); dokter.wafa@gmail.com (S.W.); wismandari01@ui.ac.id (W.W.); tri.judi@ui.ac.id (T.J.E.T.); dante.saksono@ui.ac.id (D.S.H.); pradana.soewondo@ui.ac.id (P.S.); 3 Metabolic, Cardiovascular, and Aging Cluster, The Indonesian Medical Education and Research Institute, Faculty of Medicine Universitas Indonesia, Jakarta 10430, Indonesia; calystanw@gmail.com (C.N.W.); immanuel.lix@gmail.com (I.F.); 4 Clinical Research Unit, Dr. Cipto Mangunkusumo National General Hospital, Jakarta 10430, Indonesia; 5 Division of Tropical Disease and Infection, Department of Internal Medicine, Faculty of Medicine Universitas Indonesia, Dr. Cipto Mangunkusumo National Referral Hospital, Jakarta 10430, Indonesia; robert.sinto01@ui.ac.id; 6 Division of Cardiology, Department of Internal Medicine, Faculty of Medicine Universitas Indonesia, Dr. Cipto Mangunkusumo National Referral Hospital, Jakarta 10430, Indonesia; arif.mansjoer@gmail.com

**Keywords:** ARDS, waist circumference, COVID-19 severity, obesity, poor outcome

## Abstract

(1) Background: This study aims to assess visceral fat values, waist circumference (WC), body mass index (BMI), and body fat percentage for their ability to predict poor outcomes during COVID-19 patients’ hospitalization; (2) Methods: This study was a prospective cohort of mild–moderate COVID-19 patients hospitalized at Dr. Cipto Mangunkusumo National General Hospital from December 2020 to March 2021. This study includes hospitalized patients over 18 diagnosed with COVID-19 using RT-PCR. Patients who do not have chest radiography, waist circumference, a bioimpedance analyzer (BIA) error, or are unable to stand or mobilize during the examination are excluded from this study. Cox regression was used for multivariate analysis; (3) Results: The study included two hundred sixty-one patients. The median visceral fat value was 10 (equivalent to 100 cm^2^), the WC was 93.4 cm, the BMI was 26.1 kg/m^2^, and the body fat percentage was 31.5%. Based on multivariate Cox regression, WC was statistically significant as an independent factor influencing poor outcomes in COVID-19 patients (RR 1.037 [95% CI 1.011–1.064]) along with COVID-19 degree of severity (RR 3.063 [95% CI 1.537–6.104]) and comorbidities (RR 2.123 [95% CI 1.017–4.435]); (4) Conclusions: Waist circumference can influence poor outcomes in confirmed COVID-19 patients during hospitalization.

## 1. Introduction

Obesity has been reported to be a risk factor for poor outcomes in COVID-19 patients. Obesity is associated with severe disease progression, the development of acute respiratory distress syndrome, increased rates of ICU admission, the need for invasive mechanical ventilation, and increased mortality in COVID-19 hospitalized patients [1,2,3,4]. While many low-to-middle income countries, including Indonesia, may be associated with a lower risk of severe COVID-19 infection due to its demographic composition, which is dominated by a relatively young population [5]. Uneven health infrastructure distribution and high rates of metabolic diseases, such as obesity, which affects more than 30% of the adult population, can contribute to an increased risk of severe COVID-19 [6,7].

One of the main factors in the mechanism by which obesity is associated with the adverse outcome of COVID-19 is increased pro-inflammatory adipokines [8,9,10]. Central obesity, defined by a high visceral fat distribution, is more associated with several metabolic complications, resulting in a worse outcome risk [11]. Visceral fat accumulation was more closely associated with an altered adipokine profile, which causes pro-inflammatory responses, leading to various metabolic disorders and more severe inflammatory manifestations [12]. Furthermore, obesity is a significant risk factor for metabolic dysfunction-associated fatty liver disease (MAFLD), which is linked to increased susceptibility to infections due to chronic inflammation and immune dysregulation. This condition underscores the need for vigilant infection monitoring and management in obese patients with MAFLD to reduce complications [13]. Moreover, obesity exacerbates vitamin D deficiency, a global health issue compromising metabolic, skeletal, and immune functions. Vitamin D deficiency has been linked to a higher risk of infections, including COVID-19, with emerging evidence suggesting that vitamin D supplementation may improve outcomes, though large-scale trials are still limited [14,15].

Until now, the assessment of obesity parameters with COVID-19 outcomes using a simple assessment has been limited, and there have not been many studies comparing a simple obesity assessment using a bioimpedance analyzer (BIA) to other more straightforward parameters [11]. BIA is a simple tool for assessing obesity components such as visceral fat and body fat percentage, as well as body mass index (BMI) and waist circumference (WC) [16,17].

This study aims to determine the relationship between obesity parameters, which include BMI, WC, body fat percentage, and visceral fat, and poor COVID-19 outcomes, such as ARDS and death during hospitalization of confirmed COVID-19 cases.

## 2. Materials and Methods

### 2.1. Subjects and Study Design

This study was a part of the COVID-19, Aging, and Cardiometabolic Risk Factors (CARAMEL) study, a prospective cohort study conducted in Jakarta by the Metabolic, Cardiovascular, and Aging Cluster of The Indonesian Medical Education and Research Institute Faculty of Medicine, Universitas Indonesia, to describe the cardiometabolic characteristics of COVID-19 infection patients. The detailed protocol is provided as a Supplement (Supplementary S1). This study has been approved by the Ethical Committee Board Faculty of Medicine Universitas Indonesia (KET-1112/UN2.F1/ETIK/PPM.00.02/2020, approved on 28 September 2020).

The cohort study was conducted on patients with confirmed COVID-19, aged ≥18 years, and admitted to Dr. Cipto Mangunkusumo National General Hospital, Jakarta, Indonesia, between 1 December 2020, and 31 March 2021. The diagnosis of COVID-19 was established by detecting SARS-CoV-2 RNA in nasopharyngeal swab specimens by RT-PCR testing. All patients diagnosed with confirmed COVID-19 admitted to the institution were then asked to provide written informed consent before participating in the study. This study specifically included subjects registered with mild- or moderate-severity disease according to WHO interim guidance at admission [18]. Mild-severity disease was defined as symptomatic patients who met the case definition for COVID-19 without evidence of viral pneumonia or hypoxia. Moderate-severity disease was defined as patients who met the case definition for COVID-19 and exhibited clinical signs of pneumonia (fever, cough, dyspnea, rapid breathing) but showed no signs of severe pneumonia, including SpO_2_ ≥ 90% on room air [18]. Subjects who could not stand or mobilize during the initial physical examination or had incomplete data throughout the follow-up were excluded. All patients were followed up until they had favorable or unfavorable treatment outcomes. A centralized database stores follow-up data for patient outcomes during hospitalization.

### 2.2. Data Collection

At the initial visit, all eligible participants were asked to provide demographic information such as age, sex, previous personal history, underlying diseases, and smoking history. Furthermore, clinical symptoms associated with COVID-19 were recorded, and the patient’s vital signs were measured. An automatic blood pressure monitor was used to obtain blood pressure and heart rate (Omron, Kyoto, Japan). Meanwhile, a non-contact thermometer was used to determine body temperature (Omron, Kyoto, Japan). The patient’s oxygen supplementation was recorded, and his blood oxygen saturation was measured using finger pulse oximetry (FOX-3, Elitech Technovision, Surabaya, Indonesia).

Upon admission, trained physicians performed a physical examination, including body weight, WC, and BIA. In addition, anthropometric measurements were taken on patients who did not have severe respiratory distress. Otherwise, the anthropometric measurements were obtained during a follow-up visit. Participants’ body weight and fat composition (fat percentage, muscle mass, bone mass, basal metabolic rate, metabolic age, water ratio, and visceral fat) were determined using a mobile flat scale with Bioelectric Impedance Analysis (Tanita Model BC-601, Tanita Corp, Tokyo, Japan). BIA normal values of fat percentages for men were 10–21% for 18–39 years old, 11–22% for 40–59 years old, and 13–24% for ≥60 years old; and for women, 20–34% for 18–39 years old, 21–35% for 40–59 years old, and 22–36% for ≥60 years old [19]. Meanwhile, body height was measured using a portable stadiometer (SECA Model 206, Seca GmbH Co., Hamburg, Germany). Using the body weight and height, the BMI was calculated. Furthermore, waist circumference was measured using a tape following World Health Organization guidelines [20]. (SECA Model 201, Seca GmbH Co., Hamburg, Germany).

Central obesity is defined as waist circumference ≥ 90 cm in men and ≥80 cm in women. Obesity is a body fat percentage of ≥25% in men and ≥35% in women. A national early warning score (NEWS) was used to predict patient deterioration. NEWS < 4 was classified as a low score, and ≥4 as a moderate-high score [21]. COVID-19 severity was defined by WHO interim guidance [18].

ARDS and mortality during hospitalization of COVID-19 patients were poor outcomes in this study. Therefore, ARDS was defined using the Kigali modification of the Berlin criteria [22], which included acute onset or progressive worsening of hypoxia within one week of the insult, a cut-off of SpO_2_/FiO_2_ less than or equal to 315, the absence of the need for positive end-expiratory pressure, and the presence of bilateral opacities on chest radiograph or lung ultrasound that were not primarily hydrostatic.

Statistical analysis was performed using SPSS version 20 (IBM, Armonk, NY, USA). Numerical data were presented as mean (standard deviation; SD) or median (interquartile range; IQR), and categorical data as frequency (percentage). The Kolmogorov–Smirnov test was used to determine data normality. According to the distribution of the variables, bivariate analysis was performed using the Chi-square or Fisher exact test for categorical variables and an independent *t*-test or Mann–Whitney test for continuous variables. In the bivariate analysis, variables with a *p*-value less than 0.25 were included in the multivariate analysis. Cox regression was used for multivariate analysis. The data were presented as a risk ratio (RR) with 95% confidence intervals (CI).

## 3. Results

**Figure 1:**
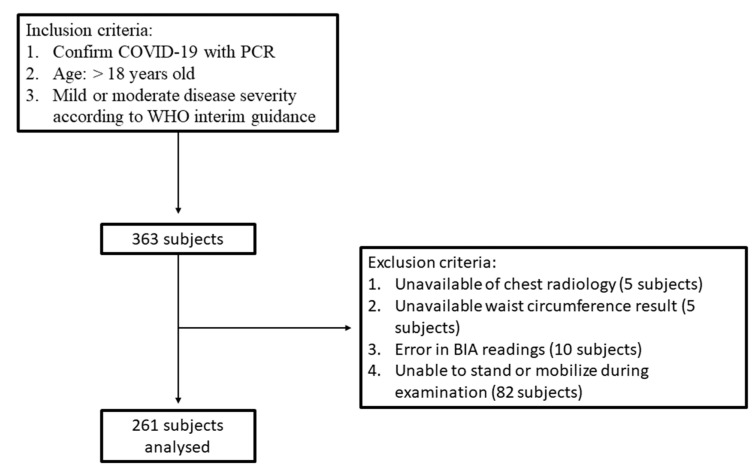
Subject recruitment process.

In our study, 261 subjects met the inclusion and exclusion criteria (Figure 1), with 82.4% falling into the 18–59 age range. The majority of the subjects (56.7%) were male, had NEWS < 4 at admission (97.3%), were diagnosed with mild disease (81.6%), were non-smokers (70.9%), and had comorbidities (52.49%), with hypertension, diabetes, and dyslipidemia being the three most common comorbidities. The median visceral fat score was 10, with 24.9% of the subjects classified as high-risk [23,24]. The average waist circumference was 93.4 cm, with 74.7% of the subjects classified as centrally obese [25]. The median BMI was 26.1 kg/m^2^, and 58.2% of the participants were obese [26]. The average body fat percentage was 31.5%, with 63.6% of the subjects obese [27]. At the follow-up, 36 (13.8%) subjects had poor outcomes. In addition, three subjects (1.2%) died who had previously had ARDS. Table 1 shows information about the subject’s baseline characteristics. Additionally, the average waist circumference and body fat percentages were analyzed separately for men and women. The average waist circumference was 94.9 cm for men and 91.5 cm for women. Similarly, the average body fat percentage was 26.0% for men and 38.7% for women (Table 2).

A bivariate analysis of the association between predictor variables and poor outcomes was performed in Table 3. It was discovered that NEWS, COVID-19 severity, comorbidities, visceral fat values, and waist circumference were statistically associated with poor outcomes during COVID-19 patient hospitalization. Further, multivariate analysis showed that waist circumference (RR 1.037 [95% CI 1.011–1.064]) could be a predictor of poor outcomes during hospitalization in mild to moderate COVID-19 patients, along with COVID-19 severity (RR 3.063 [95% CI 1.537–6.104]) and comorbidities (RR 2.123 [95% CI 1.017–4.435]) (Table 4).

## 4. Discussion

In the present study, we examined the effect of various obesity parameter indices on poor outcomes in a cohort of confirmed mild-moderate COVID-19 patients during hospitalization. According to our assessment, there were three factors associated with poor outcomes in hospitalized COVID-19 patients: waist circumference (RR 1.037, 95% CI 1.011–1.064). Moreover, we found that comorbidities (RR 2.123, 95% CI 1.017–4.435) and COVID-19 severity (RR 3.063, 95% CI 1.537–6.104) were associated with poor outcomes. These results are consistent with the previous studies [28,29,30].

In multivariate analysis, we found that waist circumference was a predictor of poor outcomes (RR 1.037, 95% CI 1.011–1.064), as was suggested by the previous studies [30,31,32,33,34,35]. In addition, waist circumference might also be more substantial when it is combined with the information on severity degree of COVID-19, NEWS at the time of hospital admission, and comorbidities, as other studies found that BMI [34], age stratification [33], hyperglycemia status [33], and gender [34] in prediction models were augmented once waist circumference was added.

Obesity is a low-grade systemic inflammation state that contributes to metabolic imbalances and alters immune responses, increasing the risk of COVID-19 progression and mortality [12]. Furthermore, increased ACE-2 expression and dysregulated activation of the renin–angiotensin–aldosterone system were found in obese individuals, indicating a role in the pathophysiology of COVID-19 [36]. Central obesity has been more closely linked to a greater number of cardiometabolic disorders [37]. Visceral adipose tissue is more metabolically active than subcutaneous adipose tissue and secretes a variety of adipokines and pro-inflammatory cytokines, including interleukin 6 (IL-6) leptin [38], which is associated with the severity of pulmonary inflammation in COVID-19 patients [39,40,41]. Furthermore, visceral obesity is linked to pro-coagulant activity and fibrinolytic suppression, which can lead to thrombotic complications and impaired ventilation restrictions, which reduce chest wall compliance and vital lung capacity, increasing pulmonary complications in COVID-19 [42].

Although initial findings identified overall obesity as a risk factor for poor progression of COVID-19, they primarily used BMI to determine obesity [43,44,45,46,47,48], which might be based on its practical aspects. Nonetheless, BMI utilization in assessing obesity might be more accurate if there is a close correlation with direct measures of obesity type that might cause a metabolic disturbance, such as waist circumference [49]. or visceral fat area [37]. Using a concurrent combination of BIA-based quantification of the visceral fat and body fat percentages with additional simple anthropomorphic measurements of waist circumference and BMI might result in a more comprehensive approach to comparing obesity phenotypes and outcomes of COVID-19 in a broader population scope. Our findings support previous findings that the central obesity phenotype is more influential in the occurrence of adverse outcomes in COVID-19 than general obesity [29,50,51,52].

However, we could not confirm the link between BMI, body fat percentage, and poor outcomes in the studied sample. There could be underlying differences between obesity phenotypes that influence outcomes. Obese patients may be at higher risk for various comorbidities, adding to the probability of severe progression in COVID-19 [53]. BMI is an indirect measure of adiposity. Simultaneously, waist circumference is commonly used as a surrogate parameter to assess visceral fat distribution and define central obesity. Blüher et al. [54]. suggested that individuals with normal BMI or body fat percentages at a possibility of obesity based on BMI or body fat percentage criteria, yet metabolically healthy, or individuals with normal BMI or body fat percentage yet have substantially elevated visceral fat values. This possibility requires further studies that directly assess central obesity and immuno-metabolic markers and their relationships with adverse outcomes in COVID-19 patients.

There is also a possibility that previous studies with a large proportion of obesity represent its higher general prevalence, especially in Europe and the United States [55], than in Asia, affecting the higher cut-off BMI criteria for obesity [56]. The meta-analysis by Pranata et al. demonstrated the association of BMI with disease severity and mortality in COVID-19 and the heterogenous pooled estimate effect [57], while the continuation meta-analysis showed low heterogeneity related to higher visceral adiposity and its association with adverse COVID-19 outcomes [53].

Furthermore, this study identified comorbidities and COVID-19 severity as risk factors for poor outcomes in COVID-19 patients. This finding was consistent with a previous study by Harbuwono et al. [58], which reported that diabetes and hypertension were associated with a worse prognosis. Diabetes and hypertension will alter adipose-resident leukocytes, causing chronic systemic inflammation [59,60,61,62,63,64]. Thus, chronic inflammation will increase ACE-2R expression. ACE-2R was the primary receptor for the spike protein of SARS-CoV-2 that facilitates its entry into human cells, leading to higher viral loads and subsequently more severe infection of COVID-19 [65,66]. Previous research has also shown that dyslipidemia is responsible for COVID-19 patients’ increased severity and mortality. COVID-19 patients with dyslipidemia have elevated LDL-C levels [67]. In response to increased oxidative stress, low-density lipoprotein (LDL) forms oxidized LDL (oxLDL) after crossing the endothelial barrier. OxLDL can form immune complexes and initiate and spread inflammatory processes. OxLDL is also a potent stimulator, capable of activating endothelial cells and monocytes and increasing the expression of numerous inflammatory proteins and receptors [68,69].

In severe COVID-19 infection, cytokine storm conditions (overproduction of inflammatory cytokines such as IL-2R, IL-6, IL-10, and TNF-α) can occur, causing vascular hyperpermeability and multiorgan failure [70]. Mudatsir et al. [71] discovered a significant increase in IL-6, CRP, and ESR levels in severe COVID-19 patients. Furthermore, other variables linked to poor outcomes, such as ferritin, lactate dehydrogenase, and procalcitonin levels, were predominantly elevated in patients with severe COVID-19. These findings supported the previous meta-analysis [72], which found that high CRP, lactate dehydrogenase, and ESR levels were associated with poor outcomes in COVID-19 patients.

The strength of this study is that our data may add to the repertoire of similar studies that require information on the differences between ethnicities or geographical regions. The causality aspect of the study is also strengthened by its prospective cohort design.

The limitations of this study include the small number of patients available for mortality assessment, as this study is part of the main study, thus not allowing further recruitment to assess mortality specifically as a sub-outcome. Additionally, the lack of standardized cut-off points for several predictor variables, such as visceral fat tissue or body fat percentage, posed challenges in categorizing obesity parameters in the analysis. The study also did not measure vitamin D levels, which may be a relevant factor. Furthermore, we did not control for variations in treatment regimens, DVT prophylaxis, or the occurrence of secondary bacterial infections, which could have influenced the outcomes.

In conclusion, we have shown that waist circumference independently contributed to the prediction of poor outcomes in COVID-19 patients, among other obesity indices. This observation accentuates the recommendation that health practitioners use waist circumference anthropometry and the severity of COVID-19 and comorbidities during hospitalization to predict poor outcomes. However, further research is needed to clarify the value of visceral fat measurement using the primary standard thoracoabdominal CT scan and validate the BIA application to assess visceral fat values in COVID-19 patients for its simplicity and economic value.

## Figures and Tables

**Table 1:** Baseline characteristics of subjects.

Variable	Total (*n* = 261)
Age (years)	
Median (IQR)	47 (32–57)
≥60, *n* (%)	46 (17.6)
18–59, *n* (%)	215 (82.4)
Sex	
Men, *n* (%)	148 (56.7)
Women, *n* (%)	113 (43.3)
NEWS	
≥4, *n* (%)	7 (2.7)
<4, *n* (%)	254 (97.3)
COVID-19 severity	
Moderate, *n* (%)	48 (18.4)
Mild, *n* (%)	213 (81.6)
Smoking status	
Active smoker, *n* (%)	20 (7.7)
Former smoker, *n* (%)	56 (21.5)
Never, *n* (%)	185 (70.9)
Comorbidities	
Without comorbidities, *n* (%)	124 (47.51)
Any comorbidities, *n* (%)	137 (52.49)
Hypertension, *n* (%)	70 (26.8)
Diabetes, *n* (%)	47 (18)
Dyslipidemia, *n* (%)	37 (14.2)
Body mass index (kg/m^2^)	
Median (IQR)	26.1 (23.1–29.4)
≥25, *n* (%)	152 (58.2%)
Waist circumference (cm)	
Mean (SD)	93.4 (12.6)
Central obesity, *n* (%)	195 (74.7)
Body fat percentage (%)	
Mean (SD)	31.5 (10.2)
Obesity	166 (63.6)
Visceral fat values	
Median (IQR)	10 (7–14)
≥15, *n* (%)	65 (24.9)
Composite outcomes, *n* (%)	36 (13.8)
ARDS, *n* (%)	33 (12.6)
Mortality, *n* (%)	3 (1.2)
Hospitalization duration (median, IQR)	9 (6–12)

Abbreviation: IQR, interquartile range; NEWS, national early warning score; SD, standard deviation; ARDS, acute respiratory distress syndrome.

**Table 2:** Average waist circumference and body fat percentage by sex in study subjects.

Variable	Sex	
	Men (*n* = 148)	Women (*n* = 113)
Waist circumference (cm)		
Mean (SD)	94.8 (13.1)	91.5 (11.6)
Body fat percentage (%)		
Mean (SD)	26.0 (8.0)	38.7 (8.0)

**Table 3:** Bivariate analysis of predictor factors of poor outcomes in hospitalized COVID-19 patients.

Variable	Poor Outcomes		*p*-Value
	Yes (*n* = 36)	No (*n* = 225)	
Age (years)			
≥60, *n* (%)	11 (23.9)	35 (76.1)	0.05
18–59, *n* (%)	25 (11.6)	190 (88.4)	
Sex			
Men, *n* (%)	26 (17.6)	122 (82.4)	0.065
Women, *n* (%)	10 (8.8)	103 (91.2)	
NEWS			
≥4, *n* (%)	4 (57.1)	3 (42.9)	0.005
<4, *n* (%)	32 (12.6)	222 (87.4)	
COVID-19 severity			
Moderate, *n* (%)	17 (35.4)	31 (64.6)	<0.001
Mild, *n* (%)	19 (8.9)	194 (91.1)	
Smoking status			
Active smoker, *n* (%)	2 (10)	18 (90)	0.78
Former smoker, *n* (%)	9 (16.1)	47 (83.9)	
Never, *n* (%)	25 (13.5)	160 (86.5)	
Comorbidities			
Any comorbidities, *n* (%)	26 (19.0)	111 (81.0)	0.01
Without comorbidities, *n* (%)	10 (8.1)	114 (91.9)	
Visceral fat values			
Median (IQR)	13.5 (9–16.7)	10 (7–14)	0.005
Waist circumference (cm)			
Mean (SD)	100.4 (90–105)	92.3 (83–100)	<0.001
Body mass index S (kg/m^2^)			
Median (IQR)	26.0 (23.9–30.8)	26.1 (22.9–29.3)	0.224
Body fat percentage (%)			
Mean (SD)	30.9 (10.7)	31.6 (10.2)	0.724

Abbreviation: IQR, interquartile range; NEWS, national early warning score; SD, standard deviation.

**Table 4:** Multivariate analysis of predictor factors for poor outcomes in hospitalized COVID-19 patients.

	Variable	RR	95% CI	*p*-Value
Model 1	Gender	1.249	0.481–3.481	0.648
	Age	1.448	0.648–3.233	0.367
	NEWS	3.374	1.039–10.953	0.043 *
	COVID-19 severity	2.700	1.330–5.481	0.006 *
	Comorbidities	1.991	0.930–4.264	0.076
	Visceral fat	1.002	0.884–1.137	0.972
	Waist Circumference	1.053	1.004–1.105	0.036 *
	BMI	0.951	0.843–1.073	0.416
Model 2	Gender	1.262	0.588–2.709	0.550
	Age	1.454	0.671–3.147	0.343
	NEWS	3.356	1.074–10.479	0.037 *
	COVID-19 severity	2.700	1.330–5.482	0.006 *
	Comorbidities	1.997	0.949–4.205	0.069
	Waist Circumference	1.054	1.007–1.103	0.025 *
	BMI	0.952	0.849–1.067	0.397
Model 3	Age	1.473	0.678–3.200	0.328
	NEWS	3.244	1.049–10.034	0.041 *
	COVID-19 severity	2.809	1.400–5.638	0.004 *
	Comorbidities	2.014	0.959–4.231	0.064
	Waist Circumference	1.056	1.010–1.104	0.016 *
	BMI	0.948	0.848–1.060	0.348
Model 4	Age	1.682	0.815–3.471	0.160
	NEWS	3.198	1.033–9.902	0.044 *
	COVID-19 severity	2.906	1.451–5.821	0.003 *
	Comorbidities	2.013	0.956–4.240	0.066
	Waist Circumference	1.038	1.012–1.064	0.004 *
Model 5	NEWS	2.879	0.947–8.752	0.062
	COVID-19 severity	3.063	1.537–6.104	0.001 *
	Comorbidities	2.123	1.017–4.435	0.045 *
	Waist Circumference	1.037	1.011–1.064	0.005 *

Abbreviation: RR, risk ratio; CI, confidence interval; NEWS, national early warning score; BMI, body mass index. * *p* < 0.05.

## Data Availability

The datasets generated during and/or analyzed during the study are available from the corresponding author on reasonable request.

## Supplementary Materials

The following supporting information can be downloaded at: https://www.mdpi.com/article/10.3390/idr16050071/s1.
