# Supplementary material for: Obesity Parameters as Predictor of Poor Outcomes in Hospitalized Patients with Confirmed Mild-to-Moderate COVID-19
Source: Infect Dis Rep. 2024 Sep 12;16(5):894–905. doi: 10.3390/idr16050071 (PMC11417886; doi:10.3390/idr16050071)
Supplement: Supplementary file 1 [file idr-16-00071-s001.zip › idr-3109369-supplementary.pdf]

Supplementary S1. Protocol paper COVID-19, Aging, and Cardiometabolic Risk Factors (CARAMEL)

1. Introduction

Coronavirus disease 2019 (COVID-19) has become a worldwide pandemic disease since March 2020. As a developing country, Indonesia is experiencing a significant impact on the healthcare system due to this situation. Although many efforts were taken to ensure disease control and management after two whole years, the mortality rate of COVID-19 in Indonesia was 2.6%, which was around two times higher than the global mortality rate.[1] This high mortality rate was thought to be associated with an increasing prevalence of obesity, diabetes, hypertension, and CVDs in Indonesia. According to Indonesian health surveys, between 2007 and 2018, obesity prevalence increased from 10.5 to 21.8, and central obesity prevalence increased from 18.8% to 31%.[2–4] In addition, the prevalence of diabetes, hypertension, and CVDs also increased, which suggests a further increase in severe cases of COVID-19.[3,4]

Recent reports describe that cardiometabolic risk factors were associated with a worse prognosis in COVID-19 patients.[5–8] Study by Harbuwono et al.[9] reported several independent risk factors for COVID-19 mortality, such as obesity, diabetes, hypertension, and CVDs. Obesity and metabolic syndrome (MetS) lead to adipose tissue inflammation and comprise a cluster of diseases associated with insulin resistance, chronic low-grade inflammation, immune system dysregulation, endothelial dysfunction, atherogenic dyslipidemia, and coagulopathy.[10] These conditions, following diabetes, hypertension, and CVDs, will alter adipose-resident leukocytes to induce chronic systemic inflammation.[11–16] Thus, chronic inflammation will increase ACE-2R expression. ACE-2R, which is abundantly found in the nasal mucous epithelium, was the primary receptor for the spike protein of SARS-CoV-2 that facilitates its entry into human cells, leading to higher viral loads and subsequently more severe infection of COVID-19.[17,18]

Another critical factor closely related to ACE-2R expression was aging. Older people are among the population with a higher proportion of ACE-2R than the general population. The ACE-2R gene is considerably associated with senescence. ACE-2 was detected in cell proliferation arrest conditions, and the ACE system was upregulated in endothelial cell senescence.[19–21] In accordance, a higher mortality rate for COVID-19 infection was observed in older age.[22–24] One retrospective study showed the low rate of ACE2-expressing cells from the nasal epithelial cell in children and its gradually enhanced expression

level in an age-dependent manner.[25] In addition, COVID-19 patients younger than 50 years with several comorbidities had a six-fold increase in the mortality rate compared to those without comorbidities.[26] These findings suggest that cardiometabolic comorbidities may be related to early aging, and the link between both factors should be elucidated to understand better early aging in the population with cardiometabolic comorbidities in COVID-19 pathophysiology.

COVID-19 infection will result in depletion of ACE2, which may lead to the decline of Angiotensin-(1-7), leading to unopposed deleterious outcomes of Angiotensin II.[27] This likely disturbs the vascular and inflammation homeostasis of the body, leading to microcirculatory derangement, endothelial damage, profound inflammation, and increased coagulopathy that characterize the more severe clinical manifestations of COVID-19 infection.[28,29] Furthermore, it was also postulated that the chronic low-grade inflammation state among the elderly and patients with cardiometabolic comorbidities leads to increased inflammatory markers (Interferon-γ and leptin) and dysregulation of the immune system. Hence, it worsens cytokine storm response, providing more severe form of the disease.[15,30–32] In addition, Chafeddine et al.[33] reported that endothelial dysfunction and severe clinical status of COVID-19 infection with a need for oxygen supplementation were independent risk factors for long COVID-19 syndrome. These findings showed a possible association between endothelial dysfunction, aging, cardiometabolic comorbidities, and long COVID-19 syndrome, and further investigations are required to characterize long COVID-19 sequelae.

The effect of age and comorbidities on severity rates and outcome of COVID-19 was likely to mirror the effects of aging, inflammatory and immune responses on almost all significant causes of mortality, underscoring the need to unravel the biological link among these factors. Aging and cardiometabolic comorbidities increase the risk for severe disease entity and mortality in COVID-19. Pathomechanisms for each of these conditions with COVID-19 have been postulated. However, the interplay between all of them and the immune system in COVID-19 needed to be elucidated promptly. This prospective observational cohort study aimed to analyze the mechanistic link among clinical and metabolic parameters, age-ing marker, endothelial dysfunction, circulating inflammatory parameters, and mucosal and systemic immune system profiles in COVID-19 patients.

CONCEPTUAL FRAMEWORK

The conceptual framework of the CARAMEL study can be found in Figure 1.

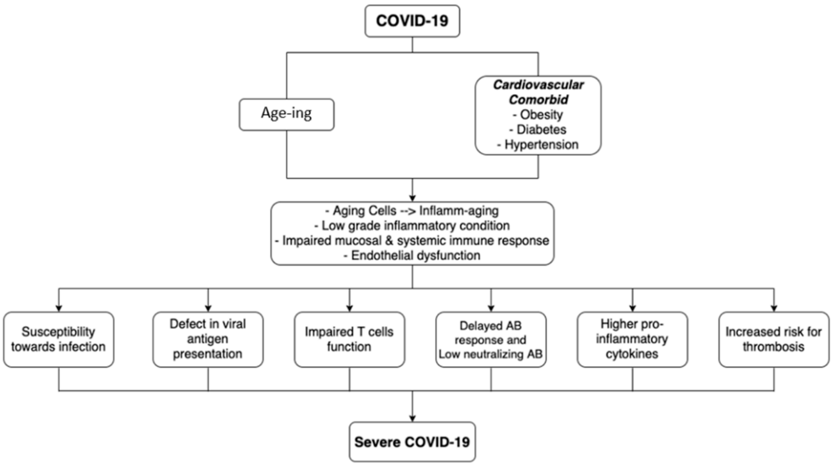

Figure 1. Conceptual Framework of the Study

2. Methods

Aims

The overall objective of this CARAMEL study is to assess the aging and cardiometabolic parameters in adult COVID-19 patients with a diverse clinical spectrum and its interrelationship with the mucosal and systemic immune profiles. This study will also try to evaluate the interplay of the parameters mentioned earlier on the incidence of long COVID syndrome.

Study design and period

A single-center observational prospective cohort study on adults (≥18 years old) with confirmed COVID-19 infection was conducted in Dr. Cipto Mangunkusumo National General Hospital, Jakarta, Indonesia, from December 2020 to December 2022. Jakarta, the capital city of Indonesia, is inhabited by many various ethnicities from all over Indonesia with different socio-economic and cultural backgrounds, being the epicenter of COVID-19 infections in Indonesia. Therefore, the implementation of research in this study area is expected to represent the diverse Indonesian population.

Study population

This study enrolled adult patients with confirmed COVID-19 diagnosis using reverse transcriptase-polymerase chain reaction (RT-PCR) from naso- and/ or oropharyngeal swab specimens admitted to the study center (Dr. Cipto Mangunkusumo National General Hospital), irrespective of their clinical disease severity. All study subjects were asked to provide written

informed consent and all necessary information/data and biological samples required for this study. Patients will be excluded if: (1) a subject deprived of freedom or subject under a legal protective measure; (2) not capable of understanding or complying with the study protocol or providing consent.

This study also recruited 4 study subsets, each consisting of 40 participants, according to their clinical symptoms and history of diabetes. These study subsets were matched for their age, sex, and body mass index (BMI). Meanwhile, participants from symptomatic COVID-19 subsets were matched for disease severity. In addition, we also recruited control subsets without COVID-19 infection, with and without type 2 diabetes (T2D) (Figure 2).

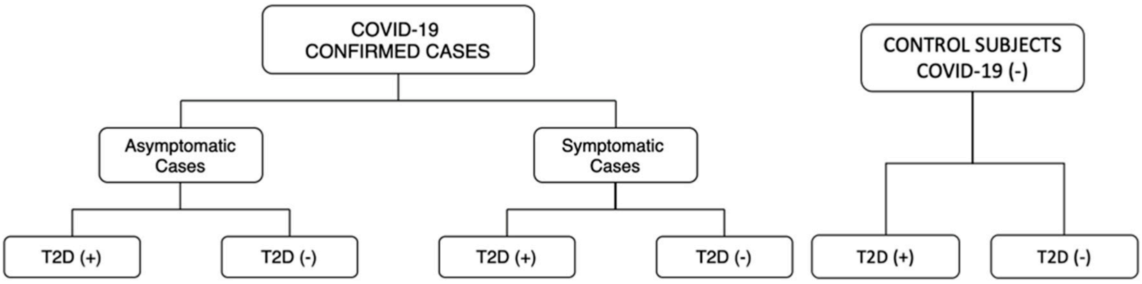

Figure 2. The subset of the Study Participants

T2D, type 2 diabetes patients

Sample size and subjects recruitment

This study aims to recruit as many as confirmed adult COVID-19 patients in the period of planned baseline measurement (December 2020–December 2021). Data of all COVID-19 patients admitted to the COVID ward each day were reported by nurse coordinators to the study coordinator. Subsequently, research teams approached and screened these potential subjects for eligibility. Information regarding this study was provided for all patients that fulfilled the eligibility criteria and proceeded with their written informed consent. Patients who agreed to give consent were continued with baseline evaluation on the same day, consisting of history taking and anthropometry measurement. The following day, all enrolled study subjects would undergo biological sample collections. The course of disease and hospitalization outcomes for each study subject was evaluated from the hospital's electronic medical records. After being discharged from the hospital, study subjects would be followed up for 12 months for the specific objective related to metabolic parameters, immunology, and long COVID syndrome.

This study also recruited an additional control group of 40 healthy participants without T2D and COVID-19 infection and 40 T2D patients without COVID-19 infection. The control group was screened and recruited from the population using flyers and other media information. The participants interested in the study will be screened and asked for written informed consent and then scheduled for a baseline evaluation, consisting of history taking, anthropometry measurement, and biological sample collections for immunology examination. The summary of the examination can be seen in Table 1.

Table 1. Overview of the Examination Procedures in the Study

**Subject Measurements**	**Timeline**				
	**T0 (0 day)**	**T1 (1 month)**	**T3 (3 months)**	**T6 (6 months)**	**T12 (12 months)**
**COVID-19 patients**					
**Clinical Parameter**	√	√	√	√	√
**Anthropometry**	√				√
**Fasting Blood Glucose (FBG)**	√	√	√	√	√
**HbA1c**	√		√	√	√
**Blood**	√	√	√	√	√
**Urine**	√	√	√	√	√
**Nasal Scrapes**	√	√			
**Nasal Brushing**	√	√			
**Non-COVID-19 Patients (DM/non-DM)**					
**Anthropometry**	√				
**Fasting Blood Glucose (FBG)**	√	√			
**HbA1c**	√				
**Blood**	√	√			
**Urine**	√	√			
**Nasal Scrapes**	√	√			
**Nasal Brushing**	√	√			
**SARS-COV-2 Rapid Antibody Testing**	√				

T0 = Baseline measurement, as soon as a confirmed diagnosis of COVID-19

T1, T3, T6, and T12 = 1, 3, 6, and 12 months after the baseline measurement

Data collection

Demographic and clinical data

At the initial visit, all eligible participants were asked to provide demographic data, such as age, sex, previous history of the disease, family history, and smoking history. Clinical symptoms related to COVID-19 were registered, and the patient's vital signs were measured. Blood pressure and heart rate were obtained using an automatic blood pressure monitor (Omron, Kyoto, Japan). Meanwhile, body temperature was obtained using a non-contact thermometer (Omron, Kyoto, Japan). Blood oxygen saturation was measured using finger pulse oximetry (Elitech Technovision, Surabaya, Indonesia), and the patient's oxygen supplementation was recorded to evaluate disease severity further.

Anthropometric measurement

Anthropometric measurements were performed for the patients without severe respiratory distress. Otherwise, the measurements were performed later during the follow-up visit. Participants' body weight and body fat composition (fat percentage, muscle mass, bone mass, basal metabolic rate, metabolic age, water ratio, and visceral fat) were measured using the mobile flat scale with Bioelectric Impedance Analysis (Tanita Model BC-601, Tanita Corp, Tokyo, Japan). Meanwhile, body height was measured using a portable stadiometer (SECA Model 206, Seca GmbH Co, Hamburg, Germany). BMI was then calculated from the body weight and body height. In addition, waist circumference was measured using measurement tape (SECA Model 201, Seca GmbH Co, Hamburg, Germany) according to World Health Organization guidelines.[34]

Blood collection

During the initial visit, fasting blood glucose was measured bedside by glucometer (Accu-check, Roche, Basel, Switzerland) using a finger prick to obtain capillary blood. Whereas fasting venous blood was collected the next day after overnight fasting in Ethylenediaminetetraacetic Acid (EDTA-K3) tube, tubes with gel separator without additives (SST), and Lithium Heparin tube. The EDTA tube was (i) used to measure HbA1c (D-10 Hemoglobin Testing System, Bio-Rad, California, USA), (ii) preserved with Genezol (Geneaid

Biotech, New Taipei City, Taiwan) for DNA and RNA isolation, (iii) centrifuged (3000 rpm, 10 minutes, RT) to obtain plasma for future research. Blood was allowed to clot in SST tubes by leaving it undisturbed at RT for 15-30 minutes, then centrifuged (3000 rpm, 10 minutes, RT) and aliquot serum in several tubes for metabolic profiles analysis. The Lithium Heparin tube was stored with CryoStor® CS10 (STEMCELL Technologies, Vancouver, Canada) for immunological profile and then centrifuged (3000 rpm, 10 minutes, RT) to obtain plasma. Whole Blood-mixed-Genezol, plasma, and serum were stored at -80ºC freezer—meanwhile, the whole blood-cryopreserved stored at liquid nitrogen temperature. In addition, for the subsets of the study, 3 extra lithium heparin tubes were collected for PBMCs isolation to study the immunological profiles described below.

Peripheral blood mononuclear cells isolation

For peripheral blood mononuclear cells (PBMC) isolation, three tubes of heparinized blood were diluted with sterile phosphate-buffered saline (PBS) (Gibco, UK) with a 1:1 ratio. Then, it was layered over the 1:1 ratio of Ficoll-paque (Sigma-aldrich, Germany) in a 50 mL-Falcon tube, and then it was centrifuged at 400 x g for 40 minutes at room temperature (RT). After centrifugation, a ringed pellet between plasma and Ficoll-paque would be formed and were pipetted to a new falcon tube. The pellets were washed twice in PBS and spun down at 400 g for 10 minutes at RT. PBMC were counted and stored in aliquots of 5-10 million cells/mL of cryomedium containing 20% dimethyl sulphoxide and 20% heat-inactivated fetal bovine serum (FBS) (Gibco, UK) at -140 °C for future research.

Nasal scrapes and nasal swab samples

Nasal scrapes were collected using a nasal mucosal curette (Rhino-Pro, Arlington) by gently scraping the inferior turbinate. Two scraps were collected from the same nostril per patient to increase cell yield. They would be deposited in an 8 mL transport medium (5 mM EDTA in PBS). Cells from nasal curettes were washed with PBS by moving back and forward the nasal curette in the fluid rapidly until the material had left scrapes; then, the supernatant was collected by centrifuging at 300 x g for 10 minutes. Cell pellets were resuspended with 1 mL PBS and split into two new tubes. One half would be fixed with paraformaldehyde (PFA), and the other half cryopreserved live.

For fixation, cell pellets from before were resuspended with PFA (final concentration will be 4% PFA), incubated for 30 minutes, collected the pellet by centrifuging at 800 x g for 10 minutes, mixed, and preserved with 1 mL cryo medium (10% DMSO in PBS), stored at 4ºC using Mr. Frosty for 10 minutes before transfer to -80ºC freezer. For live cryopreservation, cell

pellets from before were resuspended with 2 mL PBS, collected the pellet by centrifuging at 300 x g for 10 minutes, mixed and preserved with 500 uL CryoStor® CS10 (STEMCELL Technologies, Vancouver, Canada), stored at 4ºC using Mr. Frosty for 10 minutes before transfer to -80ºC freezer, and the next day moved to the liquid nitrogen tank.

The nasal swab was collected using Nasopharyngeal Specimen Collection (NEST) from both nostrils and deposited in Genezol solution for RNA isolation to study ACE2 expression of nasal mucous described below.

Study parameters

Clinical disease severity

The primary outcome of this study is the severity of the COVID-19. The severity of the disease is classified into asymptomatic, mild, moderate, and severe according to the National Institute of Health (NIH) criteria.[35] The severity of the disease then will be correlated to BMI, visceral fat, fasting blood glucose, and HbA1C. COVID-19 severity is graded according to clinical symptoms, including dyspnea and/ or shortness of breath, physical examination for oxygen saturation and respiration rate, chest imaging, and the need for oxygen supplementation. Chest imaging is taken using a mobile X-ray machine from the hospital's radiology department, which the radiology specialist from the department then analyzes.

Metabolic parameters

FBG, HbA1c, HOMA-IR, leptin, adiponectin, and L/A ratio

The study analyzed and compared changes in HOMA-IR, a surrogate marker for whole-body insulin resistance, and leptin/adiponectin (L/A) ratio between COVID-19 patients and healthy control. HOMA-IR was calculated from fasting blood glucose and fasting insulin, measured by Electro-Chemiluminescence Immunoassay (ECLIA) methods (Abbott, Abbott Park, Illinois, USA). Whereas leptin and adiponectin were measured by enzyme-linked immunosorbent assay (ELISA) method using commercial reagents (R&D systems, Minneapolis, Minnesota, USA). The leptin/adiponectin (L/A) ratio was then calculated by leptin level (ng ml^{-1}) divided by adiponectin level ($\mu g\ ml^{-1}$).

Endothelial dysfunction

Endothelial dysfunction was represented by vascular adhesion molecules—such as intercellular adhesion molecule-1 (ICAM-1) and vascular cell adhesion molecule-1 (VCAM-1). According to the manufacturer's protocol, the measurement was performed from serum samples using ELISA methods (R&D Systems, Minneapolis, Minnesota, USA).

Age-ing parameters

ACE-2 expression and telomere length were used as the aging parameter, and both parameters were measured and compared among the clinical spectrum of COVID-19 severity patients. ACE-2 expressions were obtained from nasal epithelial samples obtained using a cytology brush immediately placed into the RNA isolation kit and stored in a -80ºC freezer for 6 months. The RNA and DNA samples were then analyzed for ACE-2 expression and telomere length, respectively, using the RT-PCR method.

Immunology parameters

Systemic and nasal mucous immune profiles: Immune profiles were measured and compared between COVID-19 patients and healthy control. Systemic immune profiles were measured from whole-blood cryopreserved samples, whereas nasal mucous immune profiles were measured from cryopreserved nasal mucous swab samples. Evaluation will be performed using mass cytometry (CyTOF) with FlowJo V10 for Mac (FlowJo LLC) software.

Immune cell exhaustion: Immune cell exhaustion was measured and then compared between the spectrum of COVID-19 disease severity. The measurements were performed using flow cytometry from the cryopreserved PBMC samples. The cryopreserved PBMCs were thawed by placing the cryovial in a water bath with a temperature of 37 °C until the last ice crystals were visible. Then the warm washing medium containing 10% FBS was added. The cells were washed twice and then ready for counting. One million cells were placed into a 96-well-culture plate. They were stimulated for 6 hours with the Peptivator SARS-CoV-2 Protein_S (Miltenyi Biotec, Germany) that consisted of 15-mer sequences with 11 amino acids overlap of spike glycoprotein ("S") of SARS-CoV-2 in 1mL of culture medium (RPMI 1640 medium [Gibco, UK] supplemented with 10% FBS, 1% penicillin-streptomycin [Gibco, UK]). A protein transport inhibitor-containing Brefeldin A (BD biosciences, New Jersey, USA)—was added with the final concentration of 4 uL per 6 mL culture medium to identify intracellular markers during flow cytometry analysis. The remaining cells were stimulated with the same stimulant without a protein transport inhibitor to analyze cytokine production.

The cells were harvested and washed twice using a cell staining buffer, and then cells were fixed using a fixation buffer in the dark for 20 minutes at RT. Cells were resuspended in an intracellular staining perm wash buffer and centrifuge at 350 g for 10 minutes. Permeabilized cells were added to an optimum concentration of cocktail antibodies consisting of CD3, CD4, CD8, interferon-gamma, interleukin-2, programmed death-1 (PD-1), and CD-39 (BD biosciences, New Jersey, USA), or an appropriate negative control for 20 minutes in the dark at RT. The cells were washed twice using a staining perm wash buffer and resuspended with a cell staining buffer for flow cytometry analysis.

Pro- and anti-inflammatory cytokines: IL-6 is the pro-inflammatory cytokine while IL-10 as the anti-inflammatory cytokines were measured throughout the study and then compared between COVID-19 patients and healthy control. The measurements were performed using cytokines assay from the supernatant of the isolation PBMC method. After 6 hours of culture, cells were harvested and spun down at 350 g for 10 minutes RT. The cell's supernatants were collected and ready for cytokine analysis. IL-6 and IL-10 were measured using a bead-based multiplex assay following the manufacturer's directions.

COVID-19 antibody

COVID-19 antibody titers were measured over time and then compared between the spectrum of COVID-19 clinical severity. The antibody titers were measured from venous blood samples using the ECLIA method.

Long COVID syndrome

The proportion of patients who still presented clinical symptoms was measured and compared between the whole study participants. Evaluation of Long-COVID syndrome was asked according to clinical symptoms that are still present in the patient's follow-up visit, including fatigue, chest pain, palpitations, shortness of breath, cough, headache, anosmia, gastro-intestinal syndrome, and neuro-cognitive difficulties.[33,36]

Follow-up visit

All COVID-19 patients at baseline will be followed up four times, namely in the 1st, 3rd, 6th, and 12th months after COVID-19 infection. At 1-, 3-, 6-, and 12-months follow-up visits, all COVID-19 participants will be measured for clinical parameters, FBG, blood, and urine. Additionally, nasal scrapes and brushing will be collected only during a one-month follow-up visit, anthropometry during a 12-month follow-up visit, and HbA1c on 3-, 6-, and 12-months follow-up visits. Non-COVID-19 participants will be followed up 1 month after the baseline visit. All participants will be measured for FBG, blood, urine, and nasal brushing (Table 1).

Data management

Research data will be collected by trained research assistants and inputted into an electronic case report form using a web-based data management information system. The system uses user-level access with password protection and includes a history record for any data editing. Any form of data editing will request specific reasons to complement the good clinical practice guidelines.

Data entered will be reviewed monthly by principal researcher for data validity. After study completion, all research data will be saved into a secured Microsoft Access account

(Microsoft, Redmond, Washington, US) accessible only to authorized personnel. Meanwhile, as patients' data was saved in a medical record, no other data other than in the electronic form was used.

Statistical analysis

Categorical variables will be presented as a proportion (%); meanwhile, numerical variables will be presented as mean and standard deviation or median (interquartile range) based on their distribution. To evaluate the differences in aging, metabolic, endothelial dysfunction, thrombosis, antibody response, and inflammatory markers between the different clinical spectrums of COVID-19, we will use one-way ANOVA or Kruskal-Wallis test if not normally distributed data using the SPSS software version 25.0 (IBM; NYSE). If statistically significant, we proceed with a post-hoc test to determine which two data sets the differences lie. Furthermore, statistical analysis with a linear mixed model using R software (Rx64 version 3.5.1) and RStudio will assess the differences or changes in parameters between time points.

Meanwhile, the evaluation of the systemic and mucosal immune system will be measured using mass cytometry (CyTOF) with FlowJo V10 for Mac (FlowJo LLC) software, and the FCS files will be exported to be further analyzed with Hierarchical Stochastic Neighbor Embedding (HSNE) method in the Cytosplore framework. In addition, the cell clusters developed from the Cytosplore will be analyzed using R software (R x64 version 3.5.1) and R studio.

Patient and public involvement

Patients or the public were not involved in our research's design, conduct, reporting, or dissemination plans.

3. Discussion

The prodigious rising numbers of COVID-19 patients in Indonesia have heavily impacted the Indonesian healthcare system. The mortality rate in Indonesia is dominated by non-communicable diseases such as diabetes mellitus, heart disease, dyslipidemia, obesity, kidney disease, lung disease, and malignancy.[37] It has been reported that older age and the presence of cardiometabolic risk factors pose a poor prognostic factor of COVID-19.[19,38,39] Furthermore, CVD, hypertension, diabetes mellitus, and elevated BMI were consistently reported as significant risk factors of fatality in COVID-19 patients.[40] The CARAMEL study is the first cohort prospective study among COVID-19 patients in Indonesia, aiming to

precisely describe the phenotypic aging and cardiometabolic characteristics of patients with COVID-19 infection concerning the changes in the mucosal and systemic immune system. Particular attention will be devoted to obesity, central obesity, prediabetes, diabetes, hypertension, dyslipidemia, anti-diabetic, antihypertensive, and anti-dyslipidemia therapies.

List of Abbreviations:

CARAMEL = COVID-19, aging and cardiometabolic risk factors

ACE-2R = Angiotensin-converting enzyme-2 receptor

COVID-19 = Coronavirus disease 2019

MetS = Metabolic syndrome

RT-PCR = Reverse transcriptase-polymerase chain reaction

BMI = Body mass index

T2D = Type 2 diabetes

FBG = Fasting blood glucose

EDTA = Ethylenediaminetetraacetic acid

PBMC = Peripheral blood mononuclear cells

RT = Room temperature

FBS = Fetal bovine serum

PFA = Paraformaldehyde

NEST = Nasopharyngeal specimen collection

NIH = National institute of health

L/A = Leptin/adiponectin

ECLIA = Electro-chemiluminescence immunoassay

ELISA = Enzyme-linked immunosorbent assay

ICAM-1 = Intercellular adhesion molecule-1

CyTOF = Cytometry

HSNE = Hierarchical stochastic neighbor embedding

References

1. COVID Live - Coronavirus Statistics - Worldometer [Internet]. Worldometer. 2022. Available from: https://www.worldometers.info/coronavirus/

2. Badan Penelitian dan Pengembangan Kesehatan. Laporan Nasional Riskesdas 2007.

Lap Nas 2007 [Internet]. 2008;1–384. Available from: http://kesga.kemkes.go.id/images/pedoman/Riskesdas 2007 Nasional.pdf

3. Laporan Nasional Riskesdas. Laporan_Nasional_RKD2013_FINAL.pdf [Internet]. Badan Penelitian dan Pengembangan Kesehatan. 2013. Available from: Laporan_riskesdas_2013_final.pdf (kemkes.go.id)

4. Laporan Nasional Riskesdas. Laporan_Nasional_RKD2018_FINAL.pdf [Internet]. Badan Penelitian dan Pengembangan Kesehatan. 2018. p. 198. Available from: http://labdata.litbang.kemkes.go.id/images/download/laporan/RKD/2018/Laporan_Nasional_RKD2018_FINAL.pdf

5. Tamara A, Tahapary DL. Obesity as a predictor for a poor of COVID-19: a systemic review. Diabetes & Metabolic Syndrome: Clinical Research & Review. 2020;14(4): 655–659.

6. Agca M, Tuncay E, Yildirim E, Yildiz R, Sevim T, Ernam D, et al. Is obesity a potential risk factor for poor prognosis of covid-19? Infect Chemother. 2021;53(2):319–31.

7. Guo W, Li M, Dong Y, Zhou H, Zhang Z, Tian C, et al. Diabetes is a risk factor for the progression and prognosis of COVID-19. Diabetes Metab Res Rev. 2020;36(7):1–9.

8. Muhamad SA, Ugusman A, Kumar J, Skiba D, Hamid AA, Aminuddin A. COVID-19 and Hypertension: The What, the Why, and the How. Front Physiol. 2021;12(May):1–11.

9. Harbuwono DS, Handayani DOTL, Wahyuningsih ES, Supraptowati N, Ananda, Kurniawan F, et al. Impact of diabetes mellitus on COVID-19 clinical symptoms and mortality: Jakarta's COVID-19 epidemiological registry. Prim Care Diabetes [Internet]. 2022;16(1):65–8. Available from: https://doi.org/10.1016/j.pcd.2021.11.002

10. Wu H, Ballantyne CM. Metabolic Inflammation and Insulin Resistance in Obesity. Circ Res. 2020;126(11):1549–64.

11. Andersen CJ, Murphy KE, Fernandez ML. Impact of obesity and metabolic syndrome on immunity. Adv Nutr. 2016;7(1):66–75.

12. Agrawal M, Kern PA, Nikolajczyk BS. The Immune System in Obesity: Developing Paradigms Amidst Inconvenient Truths. Curr Diab Rep. 2017;17(10).

13. Gerriets VA, MacIver NJ. Role of T cells in malnutrition and obesity. Front Immunol. 2014;5:1–11.

14. Han JM, Levings MK. Immune Regulation in Obesity-Associated Adipose Inflammation. J Immunol. 2013;191(2):527–32.

15. Rebello CJ, Kirwan JP, Greenway FL. Obesity, the most common comorbidity in SARS-CoV-2: is leptin the link? Int J Obes. 2020;44(9):1810–7.

16. Misumi I, Starmer J, Uchimura1 T, Beck MA, Magnuson T, Whitmire JK. Obesity Expands a Distinct Population of T Cells in Adipose Tissue and Increases Vulnerability to Infection. Cell Rep. 2019;27(2):514–24.

17. Ni W, Yang X, Yang D, Bao J, Li R, Xiao Y, et al. Role of angiotensin-converting enzyme 2 (ACE2) in COVID-19. Crit Care. 2020;24(1):1–10.

18. Sungnak W, Huang N, Bécavin C, Berg M, Queen R, Litvinukova M, et al. SARS-CoV-2 entry factors are highly expressed in nasal epithelial cells together with innate immune genes. Nat Med. 2020;26(5):681–7.

19. Iannelli A, Favre G, Frey S, Esnault V, Gugenheim J, Bouam S, et al. Obesity and COVID-19: ACE 2, the Missing Tile. Obes Surg. 2020;30(11):4615–7.

20. Khemais-Benkhiat S, Idris-Khodja N, Ribeiro TP, Silva GC, Abbas M, Kheloufi M, et al. The redox-sensitive induction of the local angiotensin system promotes both premature and replicative endothelial senescence: Preventive effect of a standardized crataegus extract. Journals Gerontol - Ser A Biol Sci Med Sci. 2016;71(12):1581–90.

21. Song J, Hu B, Qu H, Wang L, Huang X, Li M, et al. Upregulation of angiotensin converting enzyme 2 by shear stress reduced inflammation and proliferation in vascular endothelial cells. Biochem Biophys Res Commun. 2020;525(3):812–8.

22. Lithander FE, Neumann S, Tenison E, Lloyd K, Welsh TJ, Rodrigues JCL, et al. COVID-19 in older people: a rapid clinical review. Age Ageing. 2020;49(4):501–515.

23. Vrillon A, Hourregue C, Azuar J, Grosset L, Boutelier A, Tan S, et al. COVID-19 in Older Adults: A Series of 76 Patients Aged 85 Years and Older with COVID-19. J Am Geriatr Soc. 2020;68(12):2735–43.

24. Bickler SW, Cauvi DM, Fisch KM, Prieto JM, Gaidry AD, Thangarajah H, et al. Age

Is Associated With Increased Expression of Pattern Recognition Receptor Genes and Ace2, the Receptor for Sars-Cov-2: Implications for the Epidemiology of Covid-19 Disease. bioRxiv [Internet]. 2020;(858):2020.06.15.134403. Available from: http://biorxiv.org/content/early/2020/06/16/2020.06.15.134403.abstract

25. Bunyavanich S, Do A, Vicencio A. Nasal Gene Expression of Angiotensin-Converting Enzyme 2 in Children and Adults. JAMA - J Am Med Assoc. 2020;323(23):2427–9.

26. Surendra H, Elyazar IR, Djaafara BA, Ekawati LL, Saraswati K, Adrian V, et al. Clinical characteristics and mortality associated with COVID-19 in Jakarta, Indonesia: A hospital-based retrospective cohort study. Lancet Reg Heal - West Pacific [Internet]. 2021;9:100108. Available from: https://doi.org/10.1016/j.lanwpc.2021.100108

27. Tikellis C, Thomas MC. Angiotensin-converting enzyme 2 (ACE2) is a key modulator of the renin angiotensin system in health and disease. Int J Pept. 2012;2012.

28. Zhang X, Zhang X, Li S, Niu S. ACE2 and COVID-19 and the resulting ARDS. Postgrad Med J. 2020;403–7.

29. Beyerstedt S, Casaro EB, Rangel ÉB. COVID-19: angiotensin-converting enzyme 2 (ACE2) expression and tissue susceptibility to SARS-CoV-2 infection. Eur J Clin Microbiol Infect Dis. 2021;40(5):905–19.

30. Michalakis K, Ilias I. SARS-CoV-2 infection and obesity: Common inflammatory and metabolic aspects. Diabetes Metab Syndr Clin Res Rev. 2020;14:469–71.

31. Cervia C, Nilsson J, Zurbuchen Y, Valaperti A, Schreiner J, Wolfensberger A, et al. Systemic and mucosal antibody responses specific to SARS-CoV-2 during mild versus severe COVID-19. J Allergy Clin Immunol. 2021;147(2):545-557.e9.

32. Peron JPS, Nakaya H. Susceptibility of the elderly to SARS-COV-2 infection: ACE-2 overexpression, shedding, and antibody-dependent enhancement (ADE). Clinics. 2020;75:1–6.

33. Charfeddine S, Ibn Hadj Amor H, Jdidi J, Torjmen S, Kraiem S, Hammami R, et al. Long COVID 19 Syndrome: Is It Related to Microcirculation and Endothelial Dysfunction? Insights From TUN-EndCOV Study. Front Cardiovasc Med. 2021;8:1–8.

34. World Health Organization. Waist circumference and waist-hip ratio: report of a WHO

expert consultation. World Health Organization. 2008.

35. National Institutes of Health. Treatment Guidelines Panel. Coronavirus Disease 2019 (COVID-19). Nih. 2021;2019.

36. Raveendran A V, Jayadevan R, Sashidharan S. Long COVID: an overview. Diabetes & Metabolic Syndrome: Clinical Research & Reviews. 2021;15(3):869–875.

37. Purnamasari D. The Emergence of Non-communicable Disease in Indonesia. Acta Med Indones. 2018;50(4):273–4.

38. Liu K, Chen Y, Lin R, Han K. Clinical features of COVID-19 in elderly patients: A comparison with young and middle-aged patients. J Infect. 2020;80:e14–8.

39. Apicella M, Campopiano MC, Mantuano M, Mazoni L, Coppelli A, Del Prato S. COVID-19 in people with diabetes: understanding the reasons for worse outcomes. Lancet Diabetes Endocrinol. 2020;8(9):782–92.

40. Kim CW, Aronow WS, Frishman WH. COVID-19 and Cardiometabolic Disease. Cardiol Rev. Cardiol Rev. 2021; Publish Ahead of Print.
